# A Low-Cost Time Transfer Receiver for Contributions to Coordinated Universal Time

**DOI:** 10.6028/jres.119.024

**Published:** 2014-11-06

**Authors:** Michael A Lombardi, Andrew N Novick, Victor S Zhang

**Affiliations:** National Institute of Standards and Technology, Boulder, CO 80305

**Keywords:** Coordinated Universal Time (UTC), Global Positioning System (GPS), International Atomic Time (TAI), time transfer

## Abstract

This paper describes a low-cost time transfer receiver that allows timing laboratories, including national metrology institutes and other designated institutions, to contribute data to the computation of Coordinated Universal Time (UTC). The time transfer receiver compares a laboratory’s local realization of UTC, to signals broadcast by Global Positioning System (GPS) satellites. It stores the measurement results in a format compatible with international standards, and sends data via the Internet to the Bureau International des Poids et Mesures (BIPM) for inclusion in the UTC computation. In addition to being inexpensive, the receiver was designed to be easy to use, allowing recently established timing laboratories to begin contributing to UTC with a minimal investment in training.

## 1. Introduction

International Atomic Time (TAI) and Coordinated Universal Time (UTC) are computed at the Bureau International des Poids et Mesures (BIPM) in Sevres, France, from clock data that are contributed by timing laboratories from around the world. At the end of 2013, 73 different timing laboratories were contributing to the TAI and UTC calculations. The measurement data were collected with several different time transfer techniques, each of which currently involves satellites [1].

The most widely implemented of these time transfer techniques is the “all-in-view” method that utilizes Global Positioning System (GPS) satellites. The all-in-view method is a variation of the common-view method, which is a well-established way to compare clocks located at remote sites. For example, two clocks that are separated by a wide geographic distance can be compared to each other by first comparing them to a common-view signal (*CVS*) that is broadcast by an independent transmitter. Simultaneous time difference measurements are made at sites A and B. The measurement at site A produces the *Clock A – CVS*, and the measurement at site B produces *Clock B – CVS*. The desired result, *Clock A – Clock B*, is obtained by subtracting the two measurements from each other. The main difference between all-in-view and common-view is that the all-in-method does not require the *CVS* source to be identical at both sites. For example, in the case of GPS, sites A and B do not need to simultaneously receive signals from the same GPS satellites. Instead, a *CVS* can be obtained at each site by averaging data from the entire group of GPS satellites that are currently in view. This allows the all-in-view method to be utilized over very long baselines, when no single satellite is in “common-view” at both sites.

The common-view time transfer technique has been implemented with a number of different types of terrestrial radio and network signals, and was described in the literature [2] long before the first GPS satellite was launched in 1978. However, since the origin of GPS, satellites have typically served as the *CVS* source, with the first common-view GPS measurement results being reported by the National Bureau of Standards (NBS), now known as the National Institute of Standards and Technology (NIST), in 1980 [3].

Beginning in the early 1980s, NBS designed and built several types of common-view time transfer receivers [4, 5]. The NBS designs were then provided to U. S. industry [6] so that the receivers could be procured by other timing laboratories. The early “NBS Type” receivers were code based and single frequency, meaning that they received the C/A (coarse acquisition) code from the GPS L1 band frequency at 1575.42 MHz, and did not receive the L2 frequency, nor did they process carrier phase data. They were also single-channel, meaning that they could track only one GPS satellite at a time. Data from each satellite were collected for 13 minutes, a period long enough to ensure that each receiver participating in the common-view comparison had a complete and current copy of the latest satellite ephemeris, which requires 12.5 minutes to download [7]. With some minor modifications, the NBS file format eventually became the standard used for the submission of time transfer data to the BIPM. Formally published in 1994 [8], the data format is known today as the Common GPS GLONASS Time Transfer Standard (CGGTTS).

In the 1990s, low-cost GPS receiver boards became commercially available as OEM (original equipment manufacturer) products. These receiver boards were also single frequency, but were multichannel and capable of tracking multiple satellites at once. Although the great majority of these products were designed for the positioning and navigation markets, a few products were optimized for timing applications. As a result, at least several national metrology institutes (NMIs) designed CGGTTS compatible receivers using eight-channel OEM boards, including the Borowiec Astrogeodymical Observatory (AOS) in Poland [9], the National Physical Laboratory (NPL) in the United Kingdom [10], and the National Institute of Metrology (NIM) in China [11]. Both the AOS and NPL receiver designs were once sold commercially, and are still used by a number of NMIs to contribute to UTC. In recent years, however, many timing laboratories have upgraded from single frequency to multi-frequency time transfer receivers. The newer receivers receive both the L1 and L2 GPS frequencies, and in some cases also receive signals from satellite constellations other than GPS. They also make it possible to utilize carrier phase techniques in addition to code techniques when processing their collected data. As a result, their combined measurement uncertainties are typically about a factor of two smaller than the uncertainties obtained with single frequency receivers. For these reasons, the single frequency models that were once sold commercially have been discontinued, and the models currently available to timing laboratories [12, 13] are typically from two to four times more expensive than their predecessors, costing as much as $40,000 USD.

The need remains, however, for a low-cost time transfer receiver with a price of less than $10,000 USD. This need is most apparent in regions such as Central and South America [14] and Africa [15], where new national timing laboratories are still being established, and where many existing laboratories have yet to contribute to UTC. These laboratories have limited financial resources. In addition, they typically maintain local time scales that are limited in both stability and accuracy. These laboratories do not currently need the highest level of time transfer performance. Their time scales can be easily characterized using a single-frequency time transfer receiver, and any performance advantage gained from the use of a more elaborate or expensive receiver is likely to be indiscernible.

To address this need, this paper presents a low-cost time transfer receiver, called the NIST TAI-1. This receiver outputs data in the CGGTTS format for contributions to the calculation of UTC. The receiver utilizes a 12-channel L1 band OEM receiver board, a time interval counter, a single board computer and passive backplane, and software that runs under the Microsoft Windows^1^ operating system. Although the receiver is based on single-frequency GPS time transfer technology that does not represent the state of the art, the receiver has been engineered to be reliable and easy to use. It features a modern touch-screen interface, and automates the process of data transfer to the BIPM. The receiver’s automated data uploads are frequent enough to support contributions both to UTC, which is published monthly with measurements reported at five-day intervals; and to Rapid UTC (UTCr), which is published weekly with measurements reported at one-day intervals [1, 16]. At this writing (July 2014), the receiver is already contributing to UTC and UTCr at one NMI, and is expected to be operational at three more South American NMIs by the end of 2014. In addition, the receiver will soon be made available by NIST through its Standard Reference Materials (SRM) program as Standard Reference Instrument (SRI) number 6004.

Section 2 describes the receiver’s hardware design, Sec. 3 describes its software design, and Sec. 4 describes the receiver’s operation. The method used to calibrate the receivers at NIST is described in Sec. 5. Finally, Sec. 6 provides a brief summary.

## 2. Receiver Hardware Design

**Fig. 1:**
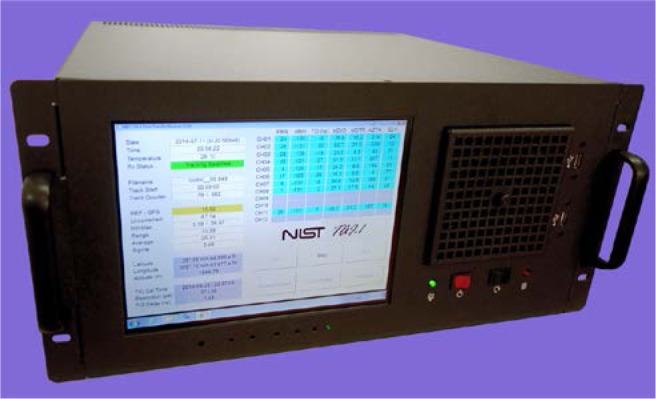
Front panel of NIST TAI-1 time transfer receiver.

**Fig. 2:**
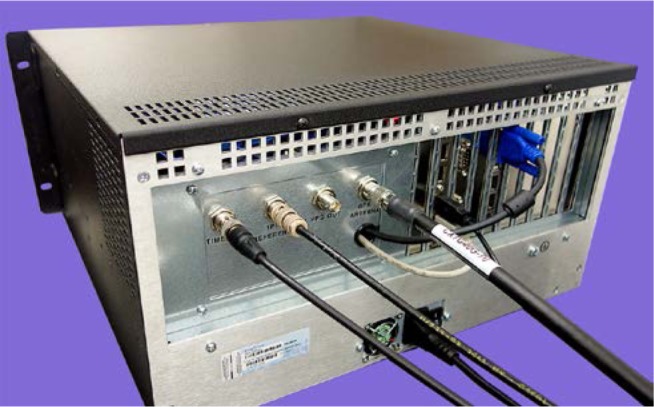
Back panel of NIST TAI-1 time transfer receiver.

The NIST TAI-1 receiver (Fig. 1) was designed with commercially-available hardware and custom software (Sec. 3) written at NIST. The unit is housed in an industrial computer chassis that includes a 264 mm diagonal (10.4 in) touch screen display with a resolution of 1024 × 768 pixels. The power supply can accept an AC input from 100 V to 240 V at either 50 or 60 Hz. The chassis includes an eight-slot passive backplane that interfaces to a single board computer, a serial interface board that mounts a 12-channel OEM GPS receiver, and a time interval counter card. The computer has 2 gigabytes of memory, utilizes a solid state drive for storage, and runs the Microsoft Windows operating system. A network interface card allows the receiver to connect to the Internet, and six universal serial bus (USB) connectors allow a variety of peripherals to be connected, including storage devices and keyboards. The back panel of the unit (Fig. 2) includes BNC connectors for a 1 pulse per second (pps) timing signal and for a 5 or 10 MHz sine wave that serves as the time interval counter time base. It also includes a TNC connector for the GPS antenna cable.

The GPS receiver is a single frequency unit (L1 band) with 12 channels. To avoid product obsolescence and to make it possible to repair and support the receivers for a number of years, the software was designed to be flexible enough to work with several different OEM receiver boards. These boards have different architectures, but have similar command sets, based on the common Motorola binary protocol. Depending upon which receiver is used, the serial communication between the receiver and computer is conducted at either 9600 or 38400 baud. These receiver modules are widely used for timing applications [17, 18] and cost less than $100 USD, roughly 2 % of the cost of the receiver modules used in the multi-frequency time transfer units [12, 13].

**Fig. 3:**
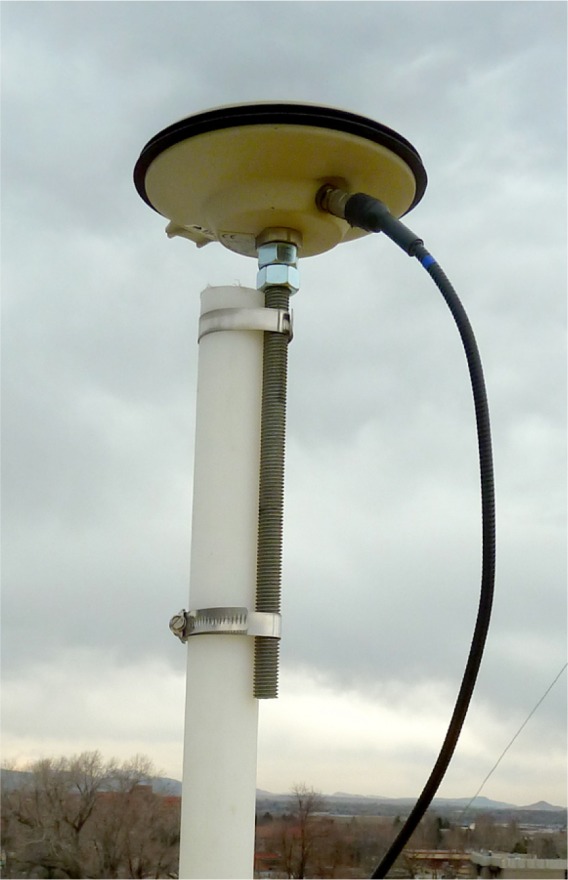
GPS antenna mounted on rooftop location.

The TAI-1 uses a GPS antenna (Fig. 3) with a “pinwheel” design [19]. These antennas are lighter, smaller, and less expensive (less than $1 000 USD) than the “choke ring” antennas used by many timing laboratories, but have proven to be equally effective at reducing the multipath signal reflections that add uncertainty to GPS time transfer measurements [20]. Low loss antenna cable is supplied, so the signal loss at the L1 frequency is only slightly more than 5 dB per 30 m of cable. The antenna includes an amplifier that is powered by 5 V dc supplied by the receiver, and that provides about 30 dB of gain. The combination of the active antenna and the low loss cable allows the receiver to work with antenna cables of up to 150 m in length, so the antenna can be located far from the laboratory when necessary.

**Fig. 4:**
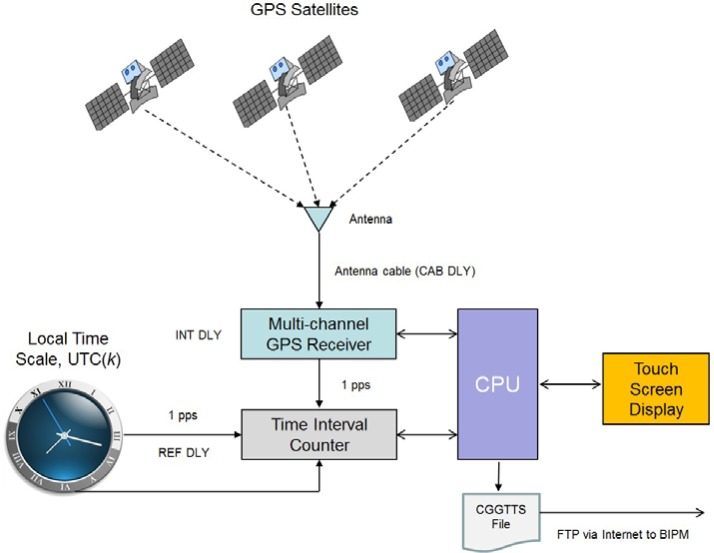
Block diagram of NIST TAI-1 time transfer receiver.

A time interval counter (TIC) is used to measure the time differences between the 1 pps signals generated by the GPS receiver and the user’s local UTC time scale, designated in BIPM parlance as UTC(*k*). The TIC is a circuit board connected to the computer’s bus via the passive backplane. As is the case with the OEM receiver boards, the software is flexible enough to be used with several different TICs, including two different NIST designs [21, 22] as well as a commercially available model. The single shot resolution of each of the various TICs that are compatible with the NIST TAI-1 receiver is less than 0.1 ns. A block diagram of the TAI-1 receiver is provided in Fig. 4.

## 3. Receiver Software Design

**Fig. 5:**
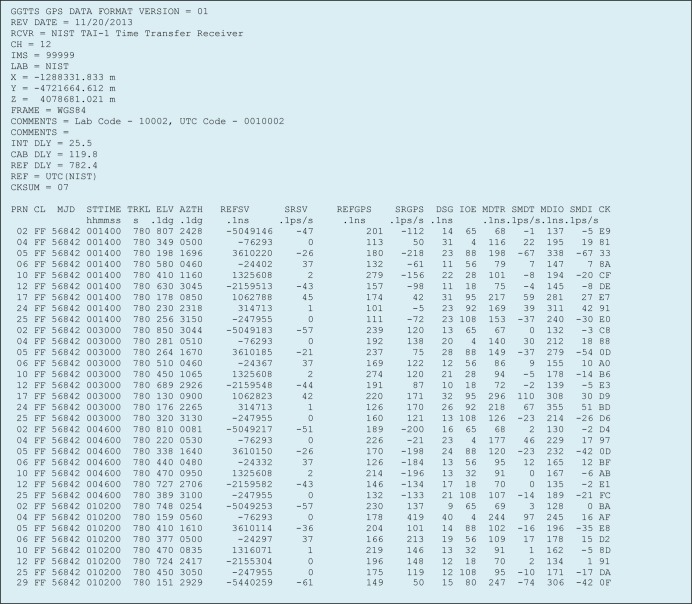
CGGTTS file created by NIST TAI-1 receiver.

The basic function of the receiver software is to measure the time differences between the individual GPS satellites and the user’s local UTC time scale once per second, and to store the measurement results and other information collected from the GPS ephemeris in a CGGTTS compatible data file (Fig. 5). A new CGGTTS file is started every day. The CGGTTS file that is currently being generated is uploaded every hour to the BIPM.

The information stored in the CGGTTS file is in ASCII (American Standard Code for Information Interchange) format. The file header [8] contains information about the receiver and the laboratory, the Cartesian (*X*, *Y*, *Z*) coordinates of the antenna, and the delay values obtained from the receiver’s calibration (Sec. 5). It also contains a hexadecimal check sum (*CKSUM*) that can be used to verify the data integrity of the file header.

The individual satellite tracks are stored in the section of the file below the header. Each satellite track is stored as one line of ASCII characters. The following paragraphs describe how the receiver software obtains the relevant fields in the satellite track, in order to achieve full compatibility with CGGTTS standard [8]. The last two characters of each satellite track contain a hexadecimal checksum (*CK*) for data verification purposes.

The *PRN* field contains the pseudo random noise code that is used to identify every satellite being tracked. The GPS constellation has 32 slots, and thus PRN codes have possible values that range from 1 to 32. Because a 12-channel GPS receiver board is used, up to 12 satellite tracks can be recorded during each data segment. The tracks recorded during each segment are sorted in the file according to their PRN numbers.

The *CL*, or class field, is a remnant from the days when receivers could track only one satellite at a time, and when the tracking schedules had to be manually entered. This field is no longer used, and is now always set to a hexadecimal value of FF.

The *MJD* field contains the Modified Julian Date, which is an integer day number obtained by computing the number of days that have elapsed between the current date and midnight on November 17, 1858, which is the MJD’s point of origin.

The *STTIME* field lists the start time of a satellite track in UTC hours, minutes, and seconds. It is important to note that the measurement data in a CGGTTS file is organized by dividing the UTC day, which consists of 1440 minutes, into 90 data segments that are each 16 minutes in length. The GPS satellite orbits, however, are based on the sidereal day, which is approximately four minutes shorter than the UTC day. Thus, one of the 90 segments is too short to record a full satellite track, so only 89 full-length satellite tracks are recorded. Due to the difference in duration between the sidereal day and the UTC day, the software must determine the start time of the first satellite track of each day, so it can determine when to open and close the daily CGGTTS files. This was accomplished by establishing a start time for the first satellite track that is four minutes later than the previous day for three consecutive days, but on the fourth day establishing a start time that is 12 minutes earlier than the previous day. As a result, the repeating sequence for the start time of the first track, in UTC hours and minutes, is 00:02, 00:06, 00:10, 00:14, 00:02, and so on. During the day, the start time for each satellite track is always 16 minutes after the start time of the previous track.

The *TRKL* field contains the duration of the satellite track in seconds. During each 16-minute data segment, only 13 minutes of data (780 s) are recorded from each satellite. Although it is no longer necessary to discard so much data with modern equipment, the standard notes that two minutes are needed “for locking onto the signal” and an additional minute is “helpful for data-processing and preparation for a new track” [8]. Thus, even though the receiver software continuously records time interval measurements during the day (with only a few seconds per track data segment used for data processing), the first two minutes of each 16-minute segment are discarded, the next 13 minutes of measurements are stored, and the last minute is also discarded. The software does not save the data unless more than 778 s of measurements were recorded. This means that the entire track will be discarded if more than one 1 pps measurement is missed.

The *ELV* and *AZTH* fields contain the elevation and azimuth angles of the satellite, respectively. Both values are stored in units of 0.1 degree. The standard indicates that they should be obtained from the “date corresponding to the midpoint of the track” [8], thus both values are obtained by averaging data collected during minute seven of the 13-minute track.

The *REFSV* field contains the satellite clock correction. The software obtains this information in one of two different ways, depending upon the OEM receiver board in use. If the receiver board allows access to the raw GPS ephemeris data, the value is computed with a second order polynomial and three coefficients obtained from subframe 1 of the ephemeris, *a_f0_*, *a_f1_*, and *a_f2_*. The *IOE* field contains an 8-bit number (a decimal value from 0 to 255) obtained from the GPS navigation message that identifies the ephemeris data set used for the computation. If the receiver board does not allow access to the raw ephemeris data, a less accurate clock correction is computed with a first order polynomial using time parameters obtained from the GPS almanac. These parameters, *a_f0_* and *a_f1_*, have less resolution and are updated less often than the time parameters in the ephemeris [23]. However, the use of the almanac data suffices, because the clock correction values are utilized only for verification purposes, and the actual time measurement data used by the BIPM for its TAI and UTC computations is contained in the *REFGPS* field.

The *REFGPS* field contains the time difference between the reference, which is the user’s local UTC time scale, and the time broadcast by the GPS satellite (identified by its PRN code). The value is recorded in units of 0.1 ns. It is obtained by collecting 52 non-overlapping data sets, each containing 15 time difference measurements (52 × 15 = 780). A least squares quadratic curve fit is applied to each data set, and the value obtained from the midpoint of the set (second eight) is obtained, reducing each 15-second data set to a single value. The resulting 52 data points are stored in an array and a least squares linear curve fit is applied. The final *REFGPS* value is obtained by using the midpoint of the least squares line, and then applying corrections for the receiver calibration (Sec. 5) and for ionospheric and tropospheric delays (discussed in the next paragraph). The *DSG* field contains the root mean square (RMS) of the linear fit residuals, expressed in units of 0.1 ns. These values provide an indication of the stability of the satellite tracks. As shown in the *DSG* column in Fig. 5, the receiver produces satellite tracks that are stable to within a few nanoseconds or less.

The ionospheric and tropospheric corrections that have been applied to the *REFGPS* value are listed in the *MDIO* and *MDTR* fields, respectively. Both corrections are modelled and based on estimates rather than measurements, hence the “MD” listed in their acronyms. They are also both processed using the method described in the previous paragraph (quadratic fit applied to 15-second data sets, linear fit applied to remaining 52 points), in accordance with the standard [8].

The model used by the receiver software to estimate the ionospheric delay was developed by Klobuchar [24, 25] and is commonly applied by nearly all single-frequency GPS receivers. Details of the algorithm are listed in [23, 24]. It utilizes the latitude, longitude, elevation angle, and azimuth as its inputs, in addition to eight coefficients obtained from the almanac broadcast by the satellites. The four “alpha” values, *α_n_*, are the coefficients for a cubic equation that represents the amplitude of the vertical delay. The four “beta” values, *β_n_* are the coefficients of a cubic equation that represents the period of the model.

**Fig. 6:**
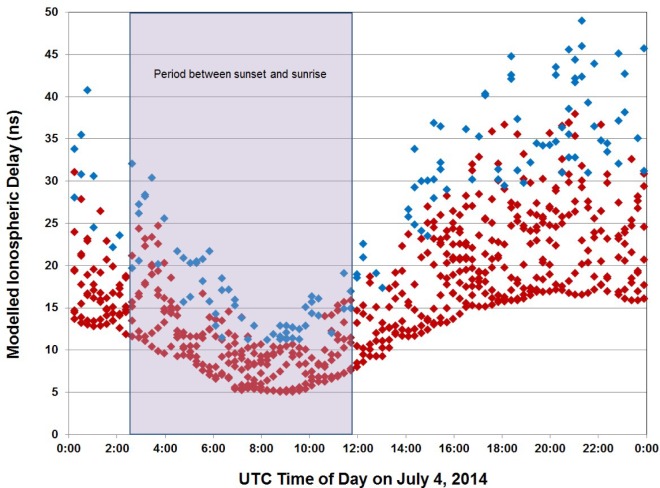
Graph of the modelled ionospheric delay correction for all satellites received in Boulder, Colorado over a 24-hour period.

At any given time of day, satellites at low elevation angles generally require a larger ionospheric delay correction than those at higher elevation angles. However, the magnitude of the correction is much larger during the daytime hours for satellites of all elevation angles than it is during the nighttime, when ionospheric delay is less of a problem. The graph in Fig. 6 shows the modelled ionospheric correction for each satellite track in a CGGTTS file collected in Boulder, Colorado on July 4, 2014 (MJD 56842). The shaded area indicates the period between sunset and sunrise when the corrections are the smallest. The blue markers indicate satellite tracks where the elevation angle was between 10° and 20° (the receiver was configured to block the reception of satellites at elevation angles below 10°). The red markers indicate satellite tracks where the elevation angle was above 20°.

The modelled estimate does not, of course, work as well as an ionospheric delay measurement, but it is expected to remove at least 50 % of the ionospheric delay [24]. Dual-frequency GPS receivers (L1 and L2 band) have the distinct advantage of being able to measure, rather than model, the ionospheric delay. When the BIPM processes CGGTTS data from a single-frequency receiver such as the TAI-1, the *MDIO* correction is removed from *REFGPS*, and a measured ionospheric delay correction (*MSIO*) is substituted, reducing the uncertainty of the *REFGPS* estimate.

GPS does not broadcast any information related to a tropospheric delay correction, but numerous models exist [26]. The TAI-1 software utilizes the tropospheric delay model originally published as a North Atlantic Treaty Organization (NATO) standard [27] and later recommended by the BIPM [28]. With this model [26, 27], the delay through the troposphere in meters, *d_trop_*, is calculated as

$$d_{trop}=d_{trop}^{z}\frac{1}{\sin(\varepsilon )+\frac{0.00143}{\tan(\varepsilon )+0.0455}},$$ (1)

where ε is the elevation angle of the satellite in radians. The $d_{trop}^{z}$ term (total zenith delay) is computed differently based on the orthometric height (altitude) of the antenna. For a height of less than 1 km,

$$d_{trop}^{z}=\left\{1430+732+\{SR\times (1-H)+0.5\Delta N(1-H^{2})\}\right\}\times 10^{-3},$$ (2)

and for a height greater than or equal to 1 km and not exceeding 9 km,

$$d_{trop}^{z}=\left\{732+\left\{\frac{N_{1}}{c}\times \exp\left(-c(H-1)\right)-\exp(-8c)\right\}\right\}\times 10^{-3},$$ (3)

where

*SR* is the global mean sea level refractivity, the recommended constant of 324.8 [27] is used in the software;

*H* is the orthometric height in kilometers;
Δ*N* = −7.32 × exp(0.005577 × *SR*);
*N*_1_ = *SR* + Δ*N*; and
$c=\frac{1}{8}\times \log(\frac{N_{1}}{105})$.

**Fig. 7:**
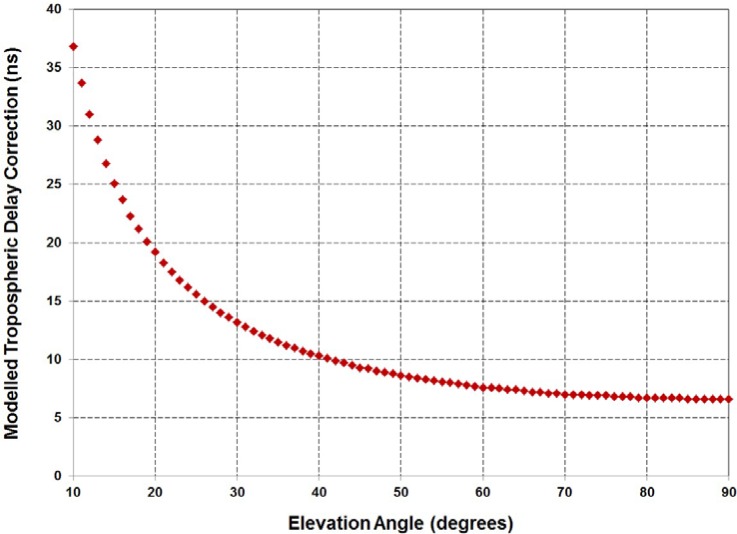
The tropospheric delay correction as a function of elevation angle in Boulder, Colorado.

The delay correction, *d_trop_* is then converted from meters to nanoseconds using the speed of light constant (299 792 458 m/s). Figure 7 shows the tropospheric delay correction as a function of elevation angles between 10° and 90° at an antenna height of 1645 m (the approximate altitude of the NIST laboratories in Boulder). Note that the correction falls below 10 ns at an elevation angle of slightly higher than 40°.

The slopes of *REFSV*, *REFGPS*, *MDTR*, and *MDIO*, each expressed in units of 0.1 ps/s, are contained in the *SRSV*, *SRGPS*, *SMDT*, and *SMDI* fields, respectively. Each of these slopes is computed from a linear fit [8].

After the receiver is started (Sec. 4), the software continues to make *REFGPS* measurements and to create new CGGTTS files (one file per day), until it is stopped by the user. Under normal operation, the receiver is expected to run continuously, 24 hours a day, 7 days a week.

## 4. Receiver Operation

Before the receiver is operated, four cables must be connected to the back panel (Fig. 2). The antenna cable is connected to the TNC connector labeled “GPS ANTENNA”. A cable with a 1 pulse per second (pps) signal from the local UTC time scale is connected to the BNC connector labeled “REFERENCE 1PPS”. The delay of this cable must be measured and recorded as it will later be entered as the *REF delay*. A cable with a stable 5 or 10 MHz signal is connected to the BNC connector labeled “TIMEBASE”. This signal can originate from the same standard as the 1 Hz signal. It serves as the time base for the time interval counter inside of the TAI-1 receiver. The signal level should be between 200 mV and 3.5 V peak-to-peak, or 70 mV to 1.25 V (rms) with 50 Ω termination. The delay of the cable used for the time base connection does not need to be measured or recorded. Finally, an Ethernet cable is used to connect the TAI-1 receiver to a network jack for Internet access.

**Fig. 8:**
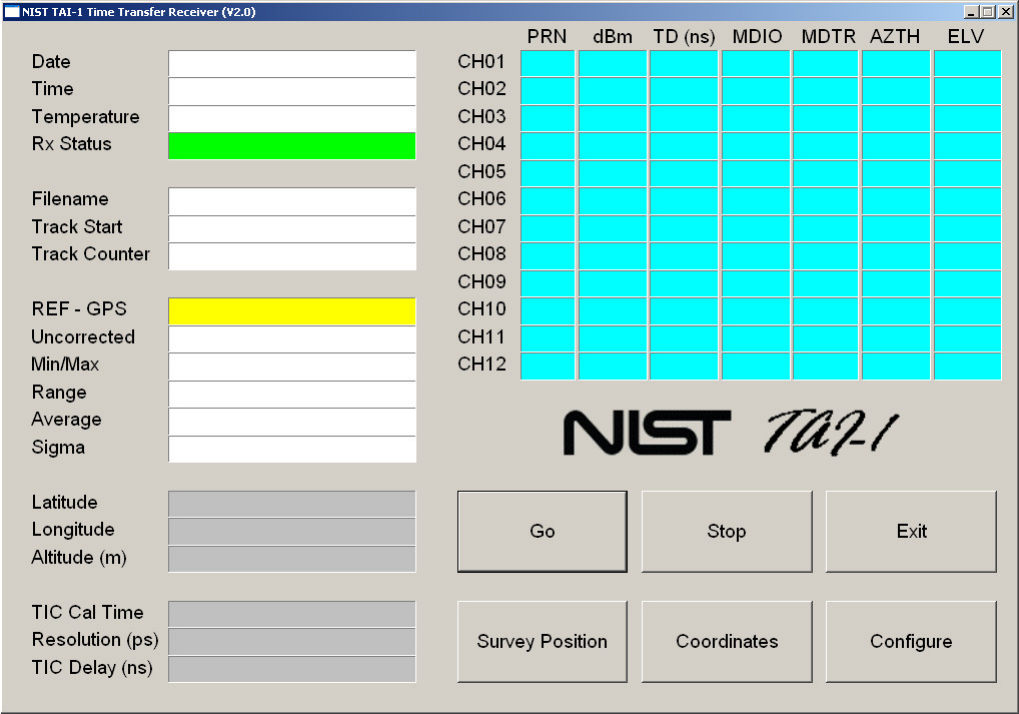
Initial receiver display.

**Fig. 9:**
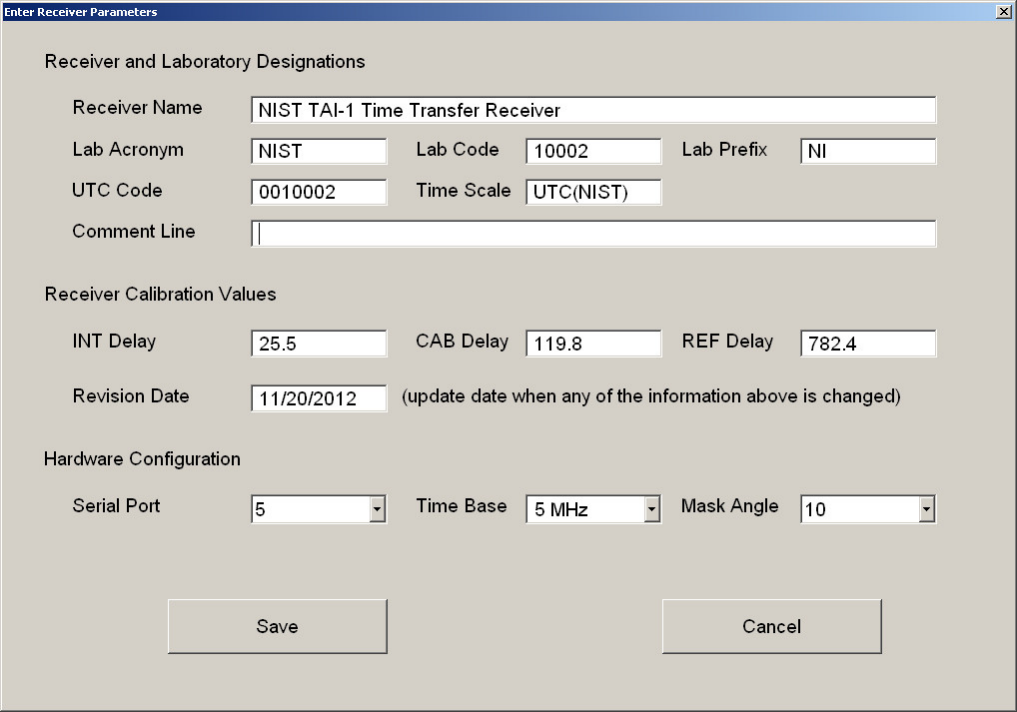
Receiver configuration screen.

After the receiver is turned on and the operating system has finished loading, the NIST TAI-1 receiver software should run automatically and the opening display will appear (Fig. 8). There are six large buttons located in the bottom right corner of the display that are used to control the receiver’s operation. The first step is to enter the configuration parameters. This is done by touching the configuration button to bring up the display shown in Fig. 9.

Some of the information on this screen was entered at NIST prior to shipment and should not be changed by the user. For example, the *Receiver Name* field contains the identifying information for the receiver (manufacturer, model, and serial number). It was entered at NIST, and is usually not changed. The *INT Delay* and *CAB Delay* were entered at NIST after the receiver was calibrated (Sec. 5). They should not be changed unless the receiver is recalibrated, either at NIST or with a travelling receiver that serves as a transfer standard. The *Serial Port* field contains the number of the serial port that controls the OEM receiver board. It should not be changed unless the receiver board is reinstalled.

The information that identifies the timing laboratory was supplied by the BIPM when the application was made to contribute to UTC. This information must be entered by the user (if it was not previously entered for the user by NIST). This information includes the *Lab Acronym* (four characters), the *Lab Code* (five digits), the *Lab Prefix* (two characters), and the *UTC Code* (seven digits). The *Time Scale* field contains the designation for your local UTC time scale. It is entered in the form UTC(*k*), where *k* is equal to the four-character laboratory acronym. For example, UTC(NIST) refers to the local UTC time scale at NIST. The *Comment Line* field allows you to enter a comment to be stored inside of all of the CGGTTS files. Entering a comment is optional.

The user is also required to enter a value in the *REF Delay* field. This value, expressed in nanoseconds, represents the delay of the cable that connects the local UTC time scale to the TAI-1 receiver. It is obtained from the measurement of the cable prior to installation. In addition, the user is allowed to select a value for the mask angle, or the lowest elevation angle above the horizon where satellites will be tracked. It can be set from 0° to 25° in 5° increments. Raising the mask angle reduces the number of visible satellites and the magnitude of the ionospheric and tropospheric corrections; lowering the mask angle does the opposite. For most locations, a mask angle of 10° is recommended. When all of the necessary information is entered, it can be saved by touching the *Save* button.

After the configuration information is saved, the user must enter the coordinates of the GPS antenna. The antenna coordinates are normally entered only once, and the process only needs to be repeated if the antenna is moved. The coordinates are entered either by keying in previously known coordinates, or by having the receiver survey it’s own antenna position for 24 hours. If the survey option is used, the latitude and longitude estimates are typically accurate to within 50 cm, but in extreme cases, the altitude error can exceed 15 m, resulting in a timing uncertainty that can approach 50 ns. Thus, the built-in antenna survey should only be used to estimate altitude if no other method is available to the user. If possible, users should independently survey the altitude of the antenna’s location with a geodetic receiver.

**Fig. 10:**
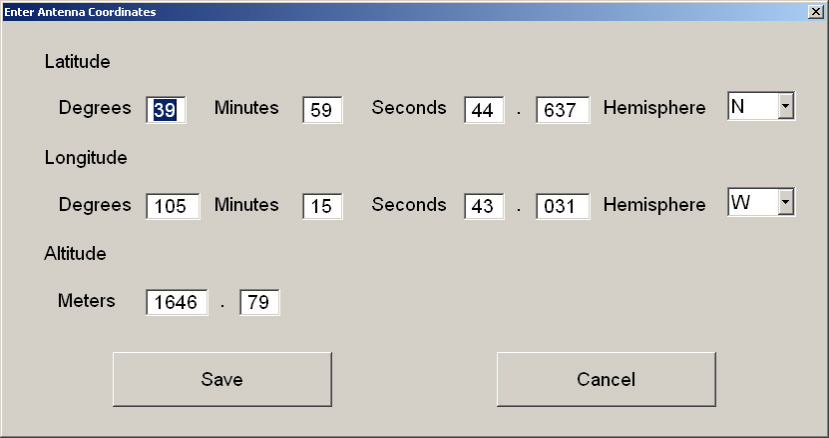
Antenna coordinates screen.

Users can manually enter the antenna coordinates by touching the *Coordinates* button. This results in a new window appearing (Fig. 10). Coordinates are then entered with an allowable resolution of 1 milliarcsecond for latitude and longitude and 1 cm for altitude, and are saved by touching the *Save* button.

Users can have the receiver survey its own antenna position by touching the *Survey Position* button. The receiver is reset, and will then begin to look for satellites. It might take several minutes before it produces its first position fix. Once the first position fix is obtained, coordinates are averaged for 24 hours (86 400 s). During the antenna survey, the latitude, longitude, and altitude fields will be updated, and the samples field will show the number of position fixes that have been averaged so far. After 86 400 valid position fixes have been obtained, the average antenna position is automatically saved and the system is ready to begin measurements.

**Fig. 11:**
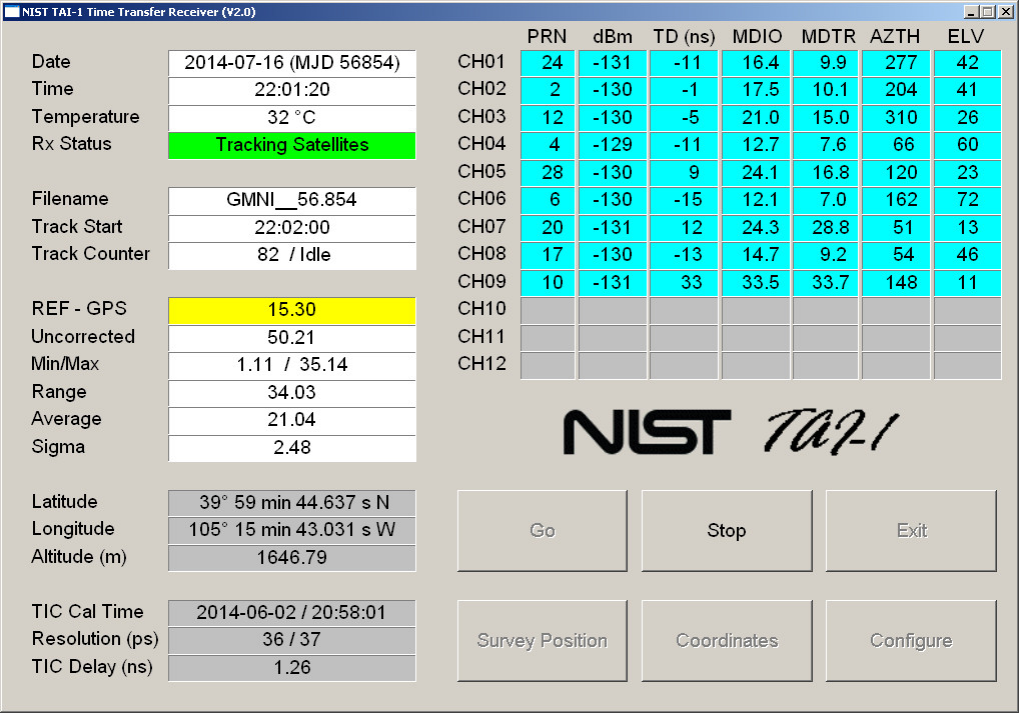
The receiver display when measurements are being recorded.

After the system parameters and antenna coordinates have been entered, the receiver can be started by simply touching the *Go* button. The receiver will then calibrate its time interval counter, check the current status of GPS satellite reception, and begin to collect and store measurements. When the receiver is recording measurements, the “Rx Status” field will be green and will display the message “Tracking Satellites”. The “REF - GPS” field will display the current time measurement against a yellow background (Fig. 11). Because time measurements should be made continuously, the user should leave the receiver running in this mode at all times. However, touching the *Stop* button will stop the measurements if it is necessary, and touching the *Exit* button will exit the software.

The receiver display is divided into two areas. The left side of the display contains general information about the receiver and the measurements (Table 1). The top right corner of the display contains information about the GPS satellites that are currently being tracked (Table 2). The rows in the top right corner of the display represent the 12 channels available for tracking GPS satellites. There are likely to be brief periods when the receiver does track 12 satellites, but it is customary for one or more of the channels to be unused at any given time.

A software application called BIPM_FTP loads and runs automatically and should always be visible on the operating system taskbar. This application sends the current CGGTTS file to the BIPM every hour via the file transfer protocol (FTP). Users are instructed to always leave BIPM_FTP running, because the data transfer to the BIPM will stop if this application is closed. Users who need to check whether the file transfers are working, or who need to change the configuration, can click on the minimized application on the taskbar to see the current upload status. Touching the *Configure* button displays a screen that allows users to enter or change the required FTP information. Login and password information is supplied by the BIPM. The application requires that ports 20 and 21 are open on the user’s local firewall, or the file transfers will be blocked.

## 5. Receiver Calibration

**Fig. 12:**
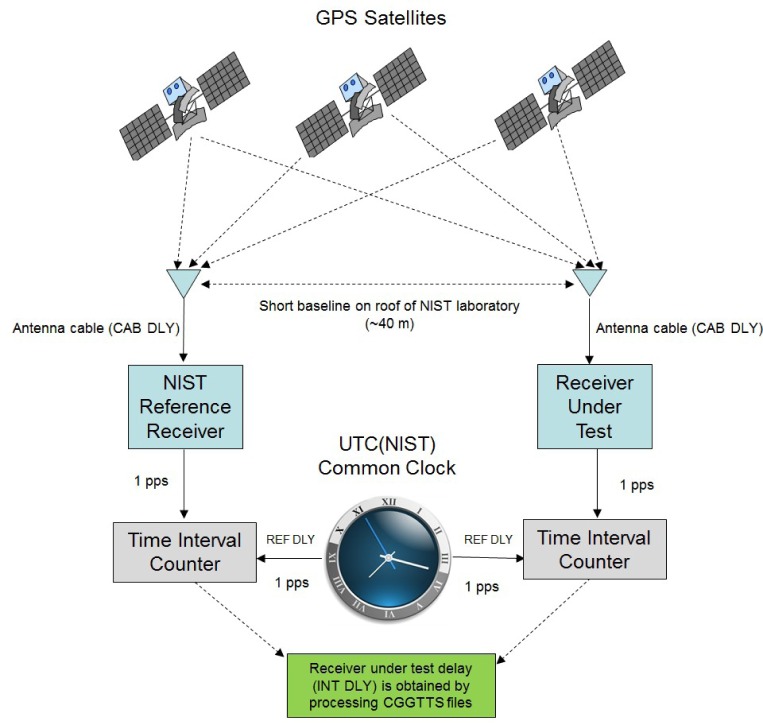
Common-clock calibration of receiver under test.

Before a TAI-1 receiver is shipped, each user is asked to supply NIST with the length of antenna cable that they need. A custom length cable is obtained, and the cable delay is calibrated at NIST with an uncertainty of about 0.1 ns. This value is keyed into the receiver under test and stored as the CAB DLY value in the CGGTTS file. The combined receiver and antenna delay, designated as INT DLY in the CGGTTS file, is obtained through a common-clock calibration, relative to the NIST reference receiver, as illustrated in Fig. 12.

The common clock is a 1 pps signal from the UTC(NIST) time scale. The cables that connect both the reference receiver and the receiver under test to the UTC(NIST) time scale are carefully calibrated by the NIST staff with an uncertainty of about 0.1 ns. The delay of the cable that connects the receiver under test to the UTC(NIST) time scale is keyed into the software and becomes the REF DLY value in the CGGTTS file.

The common-clock calibration takes place at the NIST laboratories in Boulder, Colorado. As shown in Fig. 12, the receiver under test has its antenna located just a short baseline away (~40 m) from the reference receiver antenna. The reference receiver for the calibration is a dual-frequency unit that was designated as the primary NIST time transfer receiver in 2006 [29].

**Fig. 13:**
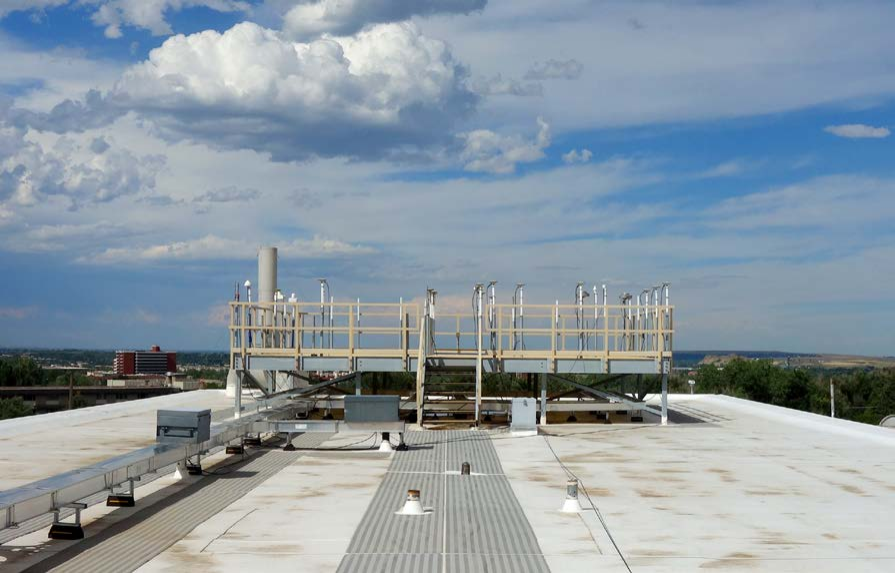
Antenna platform on rooftop of the NIST laboratories in Boulder, Colorado.

The antenna for the receiver under test is mounted on a pole on an antenna platform located on the roof of the NIST laboratories (Fig. 13). Each pole on this platform has coordinates known to within 20 cm. These coordinates were obtained through surveys conducted with geodetic receivers and a differential position service. The known coordinates are keyed in to the receiver under test prior to the calibration.

Each calibration lasts for 10 days (240 hours). A calibration is accepted only if there are no significant outliers, no signal outages or equipment interruptions, and if the time deviation, *σ_x_*(*τ*), [30] of the reduced common-view data is ~1 ns at τ = 1 day.

The results of a common-clock calibration are obtained with software written at NIST. The software processes the CGGTTS files from both the reference receiver and the receiver under test and produces the average time difference between them. This time difference is keyed into the receiver under test with a resolution of 0.1 ns. It becomes the INT DLY value in the CGGTTS file.

The uncertainty of the calibrations is limited by environmental factors, including laboratory and outdoor temperatures, but the common-clock calibration method produces stable and repeatable results. The daily values can vary by multiple nanoseconds, but historical results indicate that the measurement of the INT DLY value is likely to be stable when obtained from a 10-day average, and typically will not vary by significantly more than 1 ns [31].

**Fig. 14:**
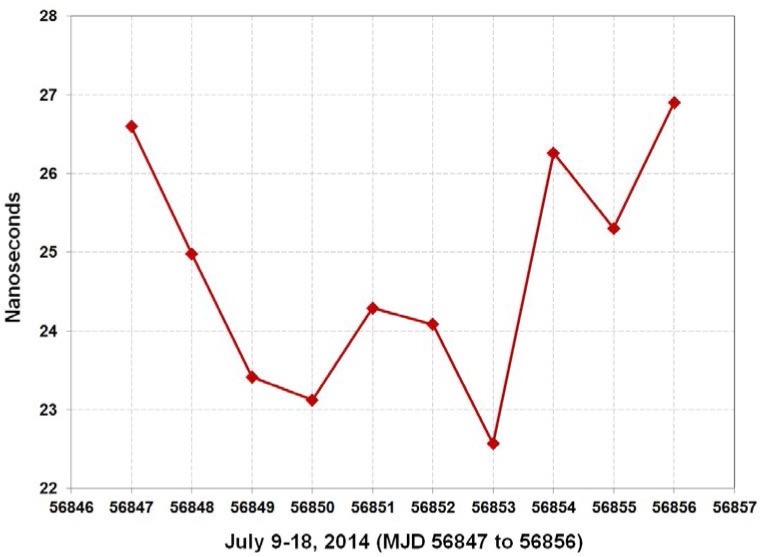
Graph of 10-day common-clock calibration of NIST TAI-1 receiver.

The REF DLY value is erased from the receiver software prior to shipment. The user is responsible for entering a new value for REF DLY that represents the delay of the cable that connects the receiver to their own time scale. Users are instructed not to change the values of CAB DLY and INT DLY, nor to change the antenna cable or antenna, as any of these changes would make the receiver calibration invalid. Figure 14 is a graph of a 10-day calibration of a NIST TAI-1 receiver, with the results summarized in Table 3.

## 6. Summary

An inexpensive time transfer receiver has been developed at NIST to allow timing laboratories to contribute data to the computation of Coordinated Universal Time (UTC). The receiver compares the laboratory’s local realization of UTC, to signals broadcast by Global Positioning System (GPS) satellites, storing results in the CGGTTS file format accepted by the Bureau International des Poids et Mesures (BIPM). The receiver was designed to fulfill a need for a low-cost time transfer receiver that can be deployed in nations where resources are limited, and where new national timing laboratories are still being established. The receiver has sufficient stability and accuracy to easily characterize the time scales of these laboratories, and has been engineered to be reliable and easy to use.

## Figures and Tables

**Table 1:** Measurement information displayed by the NIST TAI-1 receiver.

Parameter	Description
Date	The current UTC date (YYYY-MM-DD) and the Modified Julian Date.
Time	The current time (UTC HH:MM:SS) obtained from the GPS broadcast.
Temperature	The temperature obtained from a sensor on the CPU board. This sensor is read every second, but the resolution is only 1 °C.
Rx Status	This message normally changes only during GPS signal acquisition or an antenna survey. During signal acquisition, the messages are displayed with a red background. During normal operation, the “Tracking Satellites” message is displayed with a green background.
Filename	The name of the current CGGTTS file being generated by the receiver. CGGTTS filenames follow a standard format defined by the BIPM. The filename begins with “GM” to indicate that the receiver is using the GPS multichannel technique to collect data. This is followed the two-character laboratory prefix, two underscore characters, and the Modified Julian Date (MJD). The first two digits of the MJD are part of the filename and the last three digits serve as the file extension.
Track Start	The start time for the current 16-minute satellite track, as specified by the CGGTTS format. Note that data are stored for only 13 of the 16 minutes. No data are stored during the first two minutes or during the last minute of the 16-minute segment.
Track Counter	The current track number (a value from 1 to 89) and the current reading number (a value from 1 to 780). Because no data are stored during the first two minutes of the track or during the last minute, the word “Idle” is substituted for the reading number during those periods.
REF - GPS	The most recent reading from the time interval counter (displayed on a yellow background). If the 1 pps signal from the GPS receiver is early with respect to the local time scale, a negative number will be displayed. The REF – GPS reading is the time difference between the local UTC time scale and GPS, with the ionospheric and tropospheric delay corrections already applied. The unit is nanoseconds.
Uncorrected	The REF – GPS reading (above) without the ionospheric and tropospheric delay corrections. The unit is nanoseconds.
Min/Max	The smallest and largest readings recorded from the time interval counter during the current UTC day. The unit is nanoseconds.
Range	The maximum reading minus the minimum reading, in nanoseconds.
Average	The average reading recorded from the time interval counter during the current day. The unit is nanoseconds.
Sigma	The standard deviation of the difference between two successive readings, given in nanoseconds. This value is a rough estimate of the stability of the system at an averaging period of 1 second.
Latitude	The latitude of the GPS antenna. The resolution is 1 milliarcsecond.
Longitude	The longitude of the GPS antenna. The resolution is 1 milliarcsecond.
Altitude	The altitude of the GPS antenna. The resolution is 1 cm.
TIC Cal Time	The date and time of the last time interval counter calibration.
Resolution (ps)	The resolution of the start and stop inputs on the time interval counter, given in picoseconds. The resolution of both the start and stop inputs should be less than 50 picoseconds.
TIC Delay (ns)	The time offset due to delays in the time interval counter, given in nanoseconds. This value is applied as a correction to each TIC reading.

**Table 2:** Satellite information displayed by the NIST TAI-1 receiver.

Column Heading	Description
PRN	The pseudo random noise code (PRN) for each satellite being tracked. The GPS constellation has 32 slots, and thus PRN codes have possible values ranging from 1 to 32.
dBm	The signal strength of each satellite being tracked. These numbers should normally be in the −127 to −133 dBm range. Numbers smaller than −135 dBm indicate that local signal conditions are poor.
TD (ns)	The time difference (in nanoseconds) between the last time measurement recorded from the specified satellite and the average time measurement recorded from all of the satellites in view.
MDIO	The modelled ionospheric delay correction for the satellite, in nanoseconds.
MDTR	The modelled tropospheric delay correction for the satellite, in nanoseconds.
AZTH	The azimuth angle (in degrees) of the satellite.
ELV	The elevation angle (in degrees) of the satellite.

**Table 3:** Results of 10-day common-clock calibration of NIST TAI-1 receiver.

Antenna Cable	Length	21.4 m
	CAB DLY	85.3 ns
Day Number	Date (MJD)	Time Difference (ns)
1	2014-07-09 (56847)	26.6
2	2014-07-10 (56848)	25.0
3	2014-07-11 (56849)	23.4
4	2014-07-12 (56850)	23.1
5	2014-07-13 (56851)	24.3
6	2014-07-14 (56852)	24.1
7	2014-07-15 (56853)	22.6
8	2014-07-16 (56854)	26.3
9	2014-07-17 (56855)	25.3
10	2014-07-18 (56856)	26.9
Calibration Results	INT DLY (average time difference)	24.8
	Time Deviation, *σ_x_*(*τ*)	1.1

## Footnotes

1 Company and product names are mentioned for technical completeness only. No endorsement by the National Institute of Standards and Technology (NIST) is either intended or implied.
